# Self-assessment of the health status and leisure activities of individuals on haemodialysis

**DOI:** 10.1038/s41598-022-23955-7

**Published:** 2022-11-27

**Authors:** Alexandre Cardoso da Cunha, Edson Theodoro dos Santos Neto, Luciane Bresciani Salaroli

**Affiliations:** 1 Occupational Therapy Department, Federal University of Espírito Santo, Vitória, Espírito Santo Brazil; 2 Department of Social Medicine, Graduate Program in Collective Health, Federal University of Espírito Santo, Vitória, Espírito Santo Brazil; 3 Integrated Health Education Department, Graduate Program in Collective Health, Federal University of Espírito Santo, Vitória, Espírito Santo Brazil

**Keywords:** Health care, Nephrology

## Abstract

Self-assessment of health status is an important marker of social and health aspects. Haemodialysis is an option for renal replacement therapy that alters daily life and impacts social participation and the performance of tasks that give the subject a socially accepted role. In this scenario, leisure activities have the potential to generate well-being and are associated with several aspects of daily life, but few studies have analysed their relationship with the self-assessment of health status. This is a cross-sectional, census study with 1024 individuals from haemodialysis units of a Southeast Brazilian region, with the application of a questionnaire in 2019. We calculated the difference between the proportions of self-assessment of health status (positive and negative) and the two logistic regression models. The chances of individuals on haemodialysis negatively evaluating their health increase when they do not perform artistic leisure activities (OR 2.15; 95% CI 1.35–3.43), physical and sports activities (OR 3.20; 95% CI 1.86–5.52), intellectual (OR 2.21; 95% CI 1.44–3.41), manuals (OR 1.82; 95% CI 1.22–2.72), social (OR 2.74; 95% CI 1.74–4.31), tourist (OR 2.08; 95% CI 1.37–3.17) and idleness and contemplative (OR 1.92; 95% CI 1.29–2.85). Negative health self-assessment is associated with not practicing artistic, manual, physical and sporting, social, intellectual, tourist, and contemplative leisure activities, which have the function of providing social participation and giving meaning to life.

## Introduction

Self-assessment of health status has been extensively studied^1–3^ because it is a multidimensional health construct that encompasses physical and emotional components, aspects of well-being and life satisfaction^4^, in addition to sociodemographic factors, such as education^5,6^ and age^6^, lifestyle, and behaviour^7,8^, and those related to mental health^9^. It is an important marker of social participation in community^10^ and quality of life^11,12^, a predictor of morbidity^6,13^ and mortality^14^, and indicates the subjective perception in relation to health. It can also be called health perception^11,12,15^, self-reported health^16^ and subjective health^17^.

Despite the diversity of factors that the self-assessment of health status is related to, it is a very simple question about how the person assesses their own health, with the answer in the form of a Likert scale of 4 or 5 points^18^. These characteristics make it an important indicator to be studied, and there are limited studies on the subject in South America^19^.

Haemodialysis treatment for chronic kidney disease generates major changes in daily life^20^, and a lack of social support is one of the factors for individuals to determine their quality of life^21^. In these situations, for the individual on haemodialysis to better perceive their health, care should not be limited to exclusively clinical-biological issues but should also seek care that is close to the World Health Organization definition of health, which aims at complete physical, psychological and social well-being^22,23^.

The practice of leisure is a behaviour that generates well-being for the individual^24^ and helps to explain health promotion and prevention in a more comprehensive way^25^. Furthermore, we are not aware of studies that assess the self-assessment of health status associated with various leisure activities of people undergoing haemodialysis treatment. Thus, the objective of this study was to analyse the association between self-assessment of the state and leisure practices of people using haemodialysis services in a metropolitan region of south-eastern Brazil.

## Methods

### Study type and population

This study is an epidemiological, cross-sectional and census study with individuals undergoing haemodialysis treatment in the metropolitan region of a capital city in south-eastern Brazil. All users of haemodialysis services in public, philanthropic, hospitals and private clinics in the investigated region over 18 years of age of both sexes and undergoing haemodialysis renal replacement therapy between February and September 2019 participated in the research. Exclusion criteria were being under contact precautions, not residing in one of the municipalities of the metropolitan region, having been transferred to a hospital inpatient unit, and/or having limitations in understanding or answering the questions due to some acute or chronic medical condition.

### Measurements

Data on leisure activities, sociodemographic characteristics, life habits, treatment and clinical characteristics were collected. The data were categorised as follows:

1. The Self-Assessment of Health Status was assessed with the question, “In general, compared to people of your age, how do you consider your own health status?” With the answer on a 4-point Likert scale (very good, good, bad, and very bad) and categorised as “positive” (combining the answers “very good” and “good”) or “negative” (with the answers “bad” and “very bad”).
2. To measure leisure practices, the Leisure Practices Scale^26^ was used. It is a Likert-type scale that assesses eight domains of leisure: artistic (going to the movies, theatre, musical shows, participating in choir groups, etc.), manual (gardening, cooking, making crafts, woodworking, etc.), physical sports (going to the gym, playing ball, hiking, etc.), intellectual (participating in courses, reading, listening to or composing music, etc.), social (going to church, going out with friends, going to parties, visiting family, etc.), tourism (traveling, participate in excursions, take short walks in the city, etc.), virtual (browse the internet, use social networks, play video games), idleness and contemplation (appreciate nature, the sunset, meditate, etc.) with 11 points (from zero to ten, where zero means that the user never practices and ten that he always practices). The responses for each leisure domain were categorised into “no” involvement for those who do not practice leisure activities (zero response) and tertiles referring to the level of involvement. To facilitate the understanding of the analyses, the tertile with the lowest values was called “low” involvement in leisure, the tercile with intermediate values of “medium” involvement and the tercile with higher values of “high” involvement.
3. Sociodemographic characteristics: sex (“female” or “male”); age group (“18 to 29 years old”, “30 to 59 years old” or “60 years old or more”); schooling in years of study (“less than 8 years”, “9 to 11 years” or “more than 11 years”); income in minimum wage (MW) (“less than one MW”, “more than one to two MW”, “more than two to five MW” or “more than five MW”); self-reported race/colour (“white”, “black” and “brown”)^27^; marital status (“with a partner” or “without a partner”); and number of people living in the same household (“three or less” or “four or more”).
4. Characteristics of lifestyle habits: habit of consuming alcoholic beverages (“yes” or “no”); and smoking habit (“no, I never smoked”, “no, I smoked in the past, but I stopped smoking” or “yes, regularly”).
5. Clinical and treatment characteristics: type of treatment (“public”, “private” or “mixed”); time of chronic kidney disease (“less than 5 years” or “5 years or more”); time on haemodialysis (“less than 2 years” or “2 years or more”); medications used (“less than five” or “five or more”); shift that performs haemodialysis (“morning”, “afternoon” or “evening”); city of treatment and residence (“same city” or “different cities”); self-reported intradialytic complications (“none”, “one to three” or “more than three”); and self-reported illnesses (“two or less” or “three or more”).

### Data collection

Data collection took place on the premises of the haemodialysis units during the period of the permanence of the individual in the health service.

A pilot test was carried out in a test and re-test format with an interval of 15 days to assess the reliability and reproducibility of the data collection instrument. The participants were 58 individuals with CKD undergoing haemodialysis treatment in a municipality with more than 100,000 inhabitants located outside the surveyed metropolitan area. Kappa and McNemar test statistics were performed. The adjusted Kappa values for the variables of the Leisure Practices Scale ranged from 0.89 to 0.99, which expressed an almost perfect agreement, and in the McNemar tests, no variable showed a significant tendency towards disagreement at the p-value < 5% level.

### Statistical analysis

The normality of the variables was assessed using the Kolmogorov–Smirnov test. Descriptive analysis was presented in absolute and relative frequencies. Pearson’s chi-square test was used to calculate the difference between the proportions of self-rated health status (positive and negative) and the other variables. To calculate the odds ratio, a binomial logistic regression model was used for each dimension of leisure with statistical significance of up to 10% in the association test (p < 0.10), adjusting for sociodemographic variables, life habits, and clinical and treatment characteristics that were related to the self-assessment of health status. The significance level was set at 5% and the confidence interval to 95% (95% CI). Data were analysed using IBM SPSS Statistic 25.

### Ethics approval

The research was approved by the Research Ethics Committee of the Health Sciences Center of the Federal University of Espírito Santo (UFES), under number 3,002,709 and CAAE 6852817.4.0000.5060 and met the criteria of Resolution No. 466/2012 of the National Health Council. Individuals on haemodialysis who agreed to participate in the research signed the Free and Informed Consent Terms before answering the questionnaire. All the methods were carried out in accordance with relevant guidelines and regulations.

## Results

During the period of data collection, 1351 people were on haemodialysis in the haemodialysis units of a capital city in south-eastern Brazil. Of the total, 215 were excluded (137 for being in contact precautions and 78 with understanding limitations). There were 89 losses (67 due to being hospitalised in hospital units for death and seven were before data collection unit data after the beginning of unit collection in hospital units). In addition, of the 1047 eligible individuals, 23 (2.2%) refused to participate and three did not respond to the dependent variable question. Thus, 1021 individuals were included in this study.

The study population consisted predominantly of male individuals (56.7%), aged over 30 and up to 59 years (51.6%), with up to eight years of schooling (51.6%), an income between 1 and two minimum wages (44.6%), of brown race/colour (49.1%), living with a partner (55.7%), and with up to 3 people in the same household (71.1%) (Table 1). Significant differences were found for sex (p < 0.001), years of schooling (p = 0.001) and income (p = 0.001).

**Table 1:** Distribution of sociodemographic characteristics and life habits according to self-assessment of health status.

	Total	%	Self-assessment of health status				p-value*
			Positive		Negative		
			N	%	N	%	
**Sex (n = 1021)**							< 0.001
Female	443	43.3	247	55.8	196	44.2	
Male	578	56.7	386	66.8	192	33.2	
**Age group (n = 1021)**							0.774
18–29 years	59	5.8	34	57.6	25	42.4	
30–59 years	526	51.6	327	62.2	199	37.8	
60 years old or more	436	42.7	272	62.4	164	37.6	
**Schooling in years of study (n = 1010)**							0.001
≤ 8	521	51.6	296	56.8	225	43.2	
> 8 a ≤ 11	331	32.8	230	69.5	101	30.5	
> 11	158	15.6	98	62.0	60	38.0	
**Income in minimum wage**^**#**^** (n = 975)**							0.001
≤ 1	104	11.5	54	47.4	60	52.6	
> 1 e ≤ 2	440	44.6	269	61.1	171	38.9	
> 2 e ≤ 5	297	30.3	205	69.0	92	31.0	
> 5	134	13.6	86	64.2	48	35.8	
**Self-reported race/colour (n = 1008)**							0.9
White	274	27.1	183	66.8	91	33.2	
Black	240	23.8	138	57.5	102	42.5	
Brown	494	49.1	303	61.3	191	38.7	
**Marital status (n = 1021)**							0.985
With a partner	569	55.7	352	62.0	216	38.0	
Without a partner	452	44.3	281	62.0	172	38.0	
**Number of people living in the same household (n = 1019)**							0.961
≤ 3	725	71.1	450	62.1	275	37.9	
≥ 4	294	28.9	182	61.9	112	38.1	
**Habit of consuming alcoholic beverages (n = 1021)**							0.701
No	927	90.7	573	61.8	354	38.2	
Yes	94	9.3	60	63.8	34	36.2	
**Smoking habit (n = 1015)**							0.364
No, I never smoked	590	58.3	370	62.7	220	37.3	
No, I smoked in the past, but I stopped smoking	372	36.5	231	62.1	141	37.9	
Yes, regularly	53	5.2	28	52.8	25	47.2	

*Chi-square.

^#^Value of minimum wage in Brazil (September 2019): R$998.00; USD = R$4.64.

Most individuals on haemodialysis used public assistance (75.7%), had less than 5 years of chronic kidney disease (51.5%), 2 years or more of haemodialysis (77.9%), in the morning shift (41.1%), had 3 or more intradialytic complications (78.8%), self-reported having three or more diseases (68.1%), and used less than 5 medications (70.4%). For clinical and treatment variables, significant differences were found for self-reported diseases (p < 0.001) and self-reported intradialytic complications (p = 0.001; Table 2).

**Table 2:** Distribution of clinical and treatment characteristics according to self-assessment of health status.

	Total	%	Self-assessment of health status				p-value*
			Positive		Negative		
			N	%	N	%	
**Type of treatment (n = 1020)**							0.167
Public	771	75.7	473	61.3	298	38.7	
Private	224	21.9	139	62.1	85	37.9	
Mixed	25	2.4	20	80.0	5	20.0	
**Time of chronic kidney disease (n = 1016)**							0.852
< 5 anos	524	51.5	324	61.8	200	38.2	
≥ 5 anos	492	48.5	307	62.4	185	37.6	
**Time on haemodialysis (n = 964)**							0.884
< 2 years	213	22.1	131	61.5	82	38.5	
≥ 2 years	751	77.9	466	62.1	285	37.9	
**Medications used (n = 944)**							0.388
< 5	666	70.4	420	63.1	246	36.9	
≥ 5	278	29.6	167	60.1	111	39.9	
**Shift that performs haemodialysis (n = 1021)**							0.193
Morning	420	41.1	274	65.2	146	34.8	
Afternoon	360	35.4	217	60.3	143	39.7	
Evening	241	23.5	142	58.9	99	41.1	
**City of treatment and residence (n = 1021)**							0.61
Same city	639	62.7	400	62.6	239	37.4	
Different cities	382	37.3	233	61.0	149	39.0	
**Self-reported intradialytic complications (n = 1021)**							< 0.001
0	28	2.7	24	85.7	4	14.3	
≥ 1 a ≤ 3	188	18.5	143	76.1	45	23.9	
> 3	805	78.8	466	57.9	339	42.1	
**Self-reported illnesses (n = 1021)**							< 0.001
≤ 2	327	31.9	239	73.1	88	26.9	
≥ 3	694	68.1	394	56.8	300	43.2	

*Chi-Square.

For leisure activities, most individuals on haemodialysis reported not being involved in artistic activities (59%), manuals (30.8%), physical and sports activities (67.4%), tourist activities (53.3%), and virtual activities (50.5%). For intellectual activities, most of the sample reported having medium involvement (28.3%), as well as social (30.5%) and idleness and contemplative activities (28.3%). There were significant differences in artistic (p < 0.001), manual (p = 0.062), physical and sports (p < 0.001), intellectual (p < 0.001), social (p < 0.001), tourist activities (p < 0.001), virtual (p = 0.099), and idleness and contemplative activities (p = 0.001; Table 3).

**Table 3:** Relationship between involvement in leisure activities with self-assessment of health status.

	Points on the scale	Total	%	Self-assessment of health status				p-value
				Positive		Negative		
				N	%	N	%	
**Social**								< 0.001
No	0	160	15.6	81	50.63	79	49.38	
Low	1–5	302	29.7	171	56.62	131	43.38	
Medium	6–8	311	30.5	198	63.67	113	36.33	
High	9–10	248	24.2	183	73.79	65	26.21	
**Physical sports**								< 0.001
No	0	686	67.4	381	55.54	305	44.46	
Low	1–4	118	11.5	85	72.03	33	27.97	
Medium	5–7	107	10.4	77	71.96	30	28.04	
High	8–10	110	10.7	90	81.82	20	18.18	
**Tourism**								< 0.001
No	0	544	53.3	297	54.6	247	45.4	
Low	1–3	170	16.6	122	71.76	48	28.24	
Medium	4–5	142	14	94	66.2	48	33.8	
High	6–10	165	16.1	120	72.73	45	27.27	
**Manual**								0.062
No	0	313	30.8	175	55.91	138	44.09	
Low	1–5	274	26.8	177	64.6	97	35.4	
Medium	6–8	206	20.2	131	63.59	75	36.41	
High	9–10	228	22.2	150	65.79	78	34.21	
**Artistic**								< 0.001
No	0	604	59	337	55.79	267	44.21	
Low	1–4	151	14.9	104	68.87	47	31.13	
Medium	5–6	132	13	93	70.45	39	29.55	
High	7–10	134	13.1	99	73.88	35	26.12	
**Idleness and contemplation**								0.001
No	0	203	19.9	106	52.22	97	47.78	
Low	1–6	247	24.1	143	57.89	104	42.11	
Medium	7–9	288	28.3	198	68.75	90	31.25	
High	10	283	27.6	186	65.72	97	34.28	
**Intellectual**								< 0.001
No	0	239	23.4	121	50.63	118	49.37	
Low	1–5	282	27.6	176	62.41	106	37.59	
Medium	6–8	288	28.3	183	63.54	105	36.46	
High	9–10	212	20.8	153	72.17	59	27.83	
**Virtual**								0.099
No	0	516	50.5	303	58.72	213	41.28	
Low	1–5	189	18.6	124	65.61	65	34.39	
Medium	6–8	160	15.6	99	61.88	61	38.13	
High	9–10	156	15.3	107	68.59	49	31.41	

N = 1021.

Table 4 presents the model with adjustments for variables with p values less than 10% in the univariate analysis. After adjusting for variables in all domains, 7 of the 8 dimensions of leisure remained associated with self-rated health. When belonging to the group without involvement in leisure, the chances of negatively self-assessing health increased for social (OR 2.74; 95% CI 1.74–4.31), for physical and sports (OR 3.2; 95% CI 1.86–5.52), for tourist (OR 2.08; 95% CI 1.37–3.17), for manual (OR 1.8; 95% CI 1.22–2.72), for artistic (OR 2.15; 95% CI 1.35–3.43), for idleness and contemplative (OR 1.92; 95% CI 1.30–2.85), and for intellectual (OR 2.22; 95% CI 1.44–3.41). For social and intellectual activities, as involvement decreased, the chance of individuals negatively evaluating their health increased.

**Table 4:** Odds ratio and 95% confidence interval for health self-assessment by different leisure involvements.

	OR			AOR		
	p-value	OR	95% CI	p-value	AOR	95% CI
**Social**^**a**^						
No	< 0.001	2.75	(1.81–4.18)	< 0.001	2.74	(1.74–4.31)
Low	< 0.001	2.16	(1.50–3.10)	< 0.001	2.18	(1.48–3.22)
Medium	0.011	1.61	(1.12–2.32)	0.019	1.60	(1.08–2.36)
High		1			1	
**Physical sports**^**b**^						
No	< 0.001	2.20	(2.17–5.98)	< 0.001	3.20	(1.86–5.52)
Low	0.082	1.32	(0.93–3.28)	0.132	1.66	(0.86–3.23)
Medium	0.087	1.21	(0.92–3.33)	0.211	1.54	(0.78–3.02)
High		1			1	
**Tourism**^**c**^						
No	< 0.001	2.22	(1.51–3.25)	0.001	2.08	(1.37–3.17)
Low	0.844	1.05	(0.65–1.69)	0.638	0.88	(0.53–1.48)
Medium	0.215	1.36	(0.84–2.22)	0.353	1.28	(0.76–2.16)
High		1			1	
**Manual**^**d**^						
No	0.021	1.52	(1.07–2.16)	0.003	1.82	(1.22–2.72)
Low	0.78	1.05	(0.73–1.52)	0.241	1.32	(0.85–1.91)
Medium	0.632	1.10	(0.74–1.63)	0.245	1.21	(0.84–1.98)
High		1			1	
**Artistic**^**e**^						
No	< 0.001	2.24	(1.48–3.40)	0.001	2.15	(1.35–3.43)
Low	0.352	1.28	(0.76–2.14)	0.319	1.32	(0.76–2.29)
Medium	0.533	1.19	(0.69–2.03)	0.5	1.22	(0.69–2.15)
High		1			1	
**Idleness and contemplation**^**f**^						
No	0.003	1.76	(1.21–2.54)	0.001	1.92	(1.30–2.85)
Low	0.064	1.40	(0.98–1.98)	0.053	1.45	(0.99–2.11)
Medium	0.441	0.87	(0.61–1.24)	0.677	0.92	(0.63–1.35)
High		1			1	
**Intellectual**^**g**^						
No	< 0.001	2.53	(1.71–3.75)	< 0.001	2.22	(1.44–3.41)
Low	0.023	1.56	(1.06–2.30)	0.011	1.70	(1.13–2.57)
Medium	0.043	1.49	(1.01–2.19)	0.023	1.60	(1.07–2.40)
High		1			1	
**Virtual**^**h**^						
No	0.027	1.54	(1.05–2.25)	0.394	1.21	(0.78–1.86)
Low	0.558	1.15	(0.73–1.80)	0.991	1.00	(0.62–1.62)
Medium	0.211	1.35	(0.85–2.14)	0.388	1.24	(0.76–1.02)
High		1			1	

*OR* odds ratio, *AOR* adjusted odds ratio, *CI* confidence interval, *AOR* model adjusted by Sex, schooling, income, Self-reported illnesses and self-reported intradialytic complications.

Hosmer e Lemeshow test: ^a^0.734; ^b^0.305; ^c^0.857; ^d^0.968; ^e^0.733; ^f^0.882; ^g^0.723; ^h^0.993.

## Discussion

Our study is a pioneer in testing the relationship between the self-rated health status of individuals on haemodialysis and the cultural dimensions of leisure. The use of an instrument that assesses the engagement (frequency and quality) of individuals in leisure is also innovative. We found that individuals on haemodialysis who are not involved in leisure practices are more likely to have a negative self-assessment of health. This relationship occurs because leisure produces moments with the possibility of social interaction and resignification of life, which increases well-being, the perception of health and quality of life. This relationship has no concrete connection with the different leisure activities but rather with the social and subjective aspects that leisure activities provide.

The influence of leisure activities with social content on the self-assessment of health status may be related to favouring social relationships and/or associations. This study showed that the lower the involvement in social leisure activities, the greater the chances of negative self-assessments of their own health. When an individual mentions not having this type of leisure, the chances of a negative self-assessment of health can be close to three times higher. A possible explanation for this relationship is that a worse self-rated health status is associated with less social support^28^, and individuals in solitude have a negative association with leisure activities^29^. Haemodialysis requires a routine of long hours in health services and generates difficulties in maintaining leisure activities of the social type at the same frequency and intensity experienced before the beginning of treatment, which can generate unstable psychological conditions and a decrease in the quality of life^30^. The progressive increase in the chances of a negative self-assessment of health as involvement decreases can be explained by the fact that it is the cultural dimension of leisure that directly involves activities that provide associations with other individuals.

Physical and sports activities also have an impact on strengthening the social relationships of people with chronic diseases^31^ and are associated with a positive self-assessment of health status^32^. However, adherence to physical activities by individuals on haemodialysis is positively affected by motivation, anxiety, sleep quality and physical performance^33^ and is also associated with the strength of social relationships built between people^34^. In this study, we identified that individuals who reported not performing physical and sports activities were more than three times more likely to negatively evaluate their own health. The health assessment did not change for those who had some engagement with this type of leisure. This relationship indicates that the effect on the self-assessment of health status may be due to situations that favour the creation/strengthening of social bonds rather than the exercise itself.

The chances of negatively self-assessing their own health was higher among haemodialysis users who did not perform tourist activities, while no statistical differences were found between those who performed the most and other levels of engagement. This relationship can also be explained by favouring sociability. A survey carried out in Brazil pointed out that having friends or family members who encourage and make trips and trips together is an important factor in increasing the frequency of leisure activities^35^. Tourism is also carried out by people looking for experiences related to a greater purpose in life as a way of finding meaning and addressing internal problems^36^.

This search for new meanings for life and reflection on internal conflicts also explains the relationship between manual, artistic and contemplation leisure activities with the self-assessment of health status. For these leisure activities, differences were found between high and no involvement in leisure activities. Performing manual activities was related to a better perception of health, a feeling of greater control and the possibility of concrete change in the world^37^. Similarly, the relationship between artistic activities and self-assessment of health status can be explained by the association with multiple aspects of mental health and well-being^38^, in the awareness of health issues^39^ and, specifically for people on haemodialysis, are directly proportional to the perception of health they have^40^, while contemplative and leisure activities arouse positive feelings^41^. The relationship between leisure activities and health perception is known, and quality of life is an important mediator in this relationship^42^.

This study showed that the chances of negatively self-assessing health status increased as involvement in intellectual activities decreased, reaching a little more than twice as high among those who did not engage in this leisure activity. This association can be explained by studies indicating that higher levels of education are related to better health perceptions^43^. Economic, behavioural and health factors positively influence the frequency of intellectual activities throughout life, and these have a positive association with social roles developed for social inequalities^44^. The progressive increase in the chance of negatively evaluating health as the involvement with this dimension of leisure decreases can be explained by the fact that people undergoing haemodialysis, when developing more intellectual activities, discover new social functions for themselves that are not limited to the role of sick individuals.

We did not identify an association between the self-assessment of health status and involvement in the virtual activities of haemodialysis users in this study. Virtual activities occupy a place of importance in modern life and have the same characteristics as other leisure activities^45^; however, it must be considered that virtual leisure activities, when carried out in person and mediated only by technology (for example, playing video games with friends in the same real environment), arouse the positive feeling of the encounter^46^. Virtual activities allow people to star in their own stories and create other worlds, but they need experiences mediated by physical presence to consolidate bonds between individuals, overcoming an era we live in marked by alienation, individualism and loss of the sense of collectivism^47^.

Finally, this study has some limitations. This was a cross-sectional study; therefore, it was not possible to determine a causal effect between self-rated health status and leisure, but the results may support further studies that point to a causal relationship. The theme of leisure is complex, and there may be relationships with variables that were not considered in this study. However, the use of an instrument that assesses engagement and, therefore, the intensity and evaluation that each individual gives to each dimension of leisure seeks to minimise the difficulties of quantitative research on leisure that does not consider the subjective questions of the participants.

## Conclusions

The worst self-assessment of health status of individuals on haemodialysis was associated with not practicing leisure activities, regardless of clinical and treatment characteristics. The establishment of maintaining and/or expanding social relationships and building new social roles and meanings for life after starting haemodialysis treatment are important contributions that leisure activities make to improve the self-assessment of the health status. In this way, treating individuals on haemodialysis with the aim of offering complete well-being, as indicated by the World Health Organization, is a major challenge. It is important that health guidelines reinforce the importance of leisure and that public policies include the benefits of leisure practices for this population. Finally, the effectiveness of this care and the improvement of the health perception of these individuals will be greatly benefited if there are professionals in the multidisciplinary teams who can stimulate, indicate and favour leisure practices.

## Data Availability

The datasets generated and/or analyzed during the current study may be publicly available from the corresponding author after elimination of identifying information.
